# Supplementary material for: An Unusual Case of Serologically Confirmed Post-Partum Lyme Disease Following an Asymptomatic Borrelia burgdorferi Infection Acquired during Pregnancy and Lacking Vertical Transmission in Utero
Source: Pathogens. 2024 Feb 20;13(3):186. doi: 10.3390/pathogens13030186 (PMC10976031; doi:10.3390/pathogens13030186)
Supplement: Supplementary file 1 [file pathogens-13-00186-s001.zip › pathogens-2804339-supplementary.pdf]

Quest Diagnostics®

QUEST DIAGNOSTICS INCORPORATED

PATIENT INFORMATION

DOB: 03/27/1985 AGE: 24
GENDER: F

REPORT STATUS **FINAL**

ORDERING PHYSICIAN
BRODSKY E

SPECIMEN INFORMATION
SPECIMEN: 47433486

CLIENT INFORMATION
T84021 **1231018**

NONE
13:02

Test Name	In Range	Out of Range	Reference Range	Lab
LYME DISEASE AB (IGG),WB				TBR
**LYME DISEASE INTERP (IGG)**		**Positive ***	Negative	
**18 KD (IGG) BAND**		**Reactive ***		
**23 KD (IGG) BAND**		**Reactive ***		
**28 KD (IGG) BAND**		**Reactive ***		
30 KD (IGG) BAND	Nonreactive			
**39 KD (IGG) BAND**		[illegible] *****		
**41 KD (IGG) BAND**		**Reactive ***		
**45 KD (IGG) BAND**		**Reactive ***		
**58 KD (IGG) BAND**		**Reactive ***		

IgG Western Blots which have 5 (or more) of the 10 significant bands are considered positive for specific antibody to B. burgdorferi. (Proceedings of the 2nd Conf. on Lyme Disease, Dearborn, MI, 1994.)

Test Name	In Range	Out of Range	Reference Range	Lab
LYME DISEASE (IGM),WB				TBR
**LYME DISEASE INTERP (IGM)**		**Positive ***	Negative	
**23 KD (IGM) BAND**		**Reactive ***		
39 KD (IGM) BAND	Nonreactive			
**41 KD (IGM) BAND**		**Reactive ***		

IgM Western Blots which have 2 (or more) of the 3 significant bands are considered positive for specific antibody to B. burgdorferi. (Proceeding of the 2nd Conf. on Lyme Disease, Dearborn, MI, 1994.)

PERFORMING LABORATORY INFORMATION:

TBR Quest Diagnostics One Malcolm Avenue Teterboro NJ 07608 Laboratory Director: William E. Tarr, M.D.
CLIA No: 31D0696246
